# A SVM and SLIC Based Detection Method for Paddy Field Boundary Line

**DOI:** 10.3390/s20092610

**Published:** 2020-05-03

**Authors:** Yanming Li, Zijia Hong, Daoqing Cai, Yixiang Huang, Liang Gong, Chengliang Liu

**Affiliations:** School of Mechanical Engineering, Shanghai Jiao Tong University, Shanghai 200000, China; hongzj@sjtu.edu.cn (Z.H.); caidaoqing@sjtu.edu.cn (D.C.); huang.yixiang@sjtu.edu.cn (Y.H.); gongliang_mi@sjtu.edu.cn (L.G.); chlliu@sjtu.edu.cn (C.L.)

**Keywords:** field boundary line detection, vision in agriculture, support vector machine line detection, superpixel segmentation algorithm

## Abstract

Visual based route and boundary detection is a key technology in agricultural automatic navigation systems. The variable illumination and lack of training samples has a bad effect on visual route detection in unstructured farmland environments. In order to improve the robustness of the boundary detection under different illumination conditions, an image segmentation algorithm based on support vector machine was proposed. A superpixel segmentation algorithm was adopted to solve the lack of training samples for a support vector machine. A sufficient number of superpixel samples were selected for extraction of color and texture features, thus a 19-dimensional feature vector was formed. Then, the support vector machine model was trained and used to identify the paddy ridge field in the new picture. The recognition F1 score can reach 90.7%. Finally, Hough transform detection was used to extract the boundary of the ridge field. The total running time of the proposed algorithm is within 0.8 s and can meet the real-time requirements of agricultural machinery.

## 1. Introduction

Different types of automation systems for farm vehicles have been invented and introduced in agricultural fields [1,2]. Although autonomous agricultural vehicles using global positioning systems (GPS) have been in use for many years [3,4], when the satellite signals are poor, or the farmland environment is dynamic, visual environment recognition and navigation become key means for intelligent agricultural machinery navigation [5,6,7], and the vision-based navigation route detection algorithm is the core content. The navigation route mainly includes the crop line formed by the cultivated crops and the boundary line of the farmland.

The vision-based navigation route detection algorithm mainly includes two stages, image segmentation and route extraction. Earlier researchers used monochrome cameras sensitive to near-infrared spectroscopy to acquire images [8,9,10,11,12], because in the near-infrared spectroscopy crops performed more clearly with respect to the color value of the land and visually brighter. It is relatively easy to separate crops from land. With the development of color cameras, researchers have found that there is a large difference between crops and land in the green color channel. The difference in green color values can effectively divide crops and land. Compared with monochrome cameras, the segmentation algorithm based on green color difference is more robust to the changes of natural light and shadows in the image [13,14]. However, during the fallow period of harvesting or sowing, the color of crops is no longer green, but more brown and black. The algorithm based on green color difference segmentation is difficult to distinguish between crops and land. In response to this problem, some researchers have proposed that crop rows show periodic changes in the image, and crop rows are at the peak of periodic changes. By searching for pixel values of periodic changes in the image, the farmland image is segmented to determine the position of crop rows in the image [15,16]. Meanwhile, the farmland image segmentation algorithm is susceptible to natural light, researchers have reduced the effect of light on the image by converting the original image to an invariant light map [17]. After the crops and land are segmented, the routes are determined by fitting straight lines in the segmentation map. There are many ways to fit straight lines, including Hough transform [8], linear regression [9], fixed template matching [12], or random sampling consistent fitting (RANSAC) [18]. However, when there are many weeds in the farmland, straight line fitting in the binary segmentation map of green plants will cause a lot of “wrong lines”. Aiming at the problem of straight line misdetection caused by overgrown weeds, Bossu et al. [14] used wavelet transform to analyze the image in the frequency domain, and then locate the crop rows to avoid errors caused by straight line fitting.

Obviously, in the course of route detection, the core algorithm is image segmentation. During image segmentation, the above-mentioned route detection algorithm based on color difference is a detection algorithm based on models and knowledge, while the field boundary detection algorithm based on machine learning is a training learning method based on feature data. It is used in different lighting conditions and different detection conditions. With sufficient training samples, through appropriate feature extraction and selection, machine learning algorithms can obtain accurate classification results of different parts based on feature data, and the classification results are less affected by natural light. With the improvement of hardware computing power, machine learning algorithms have been applied in machine vision [19].

This paper proposes a paddy field boundary detection algorithm based on machine learning. A simple linear iterative clustering algorithm (SLIC) [20] was used to perform superpixel segmentation preprocessing of paddy field images, extract color features and texture features of superpixels, and form a 19-dimensional feature vector as the input of a support vector machine (SVM) [21]. The trained SVM model was used to classify different blocks in the image and segment the image. Then, the Hough transform was used to extract the paddy field boundary.

## 2. Materials and Methods

A ZED camera was installed on a Yanmar VP6E paddy field broadcast machine. The camera collected pictures at a resolution of 1280 × 720 and at a rate of 30 frames per second. Color images were obtained mainly in the paddy field in Pudong District, Shanghai and Songjiang District, Shanghai.

### 2.1. SLIC Superpixel Segmentation for Paddy Field Pictures

The superpixel segmentation of SLIC paddy field pictures is divided into two steps:

1. Initialize the paddy field image clustering center.

Set the number of cluster centers to *k* and the vector value of the *i*-th cluster center is ***C****_i_*(*l_i_*, *a_i_*, *b_i_*, *x_i_*, *y_i_*), where (*l_i_*, *a_i_*, *b_i_*) is the channel value of the cluster center in the CIELAB color space and (*x_i_*, *y_i_*) is the coordinate of the cluster center in the image. The initialized cluster centers are evenly distributed in step length *S* (Equation (1)).

(1) $$S=\sqrt{N/k}.$$

Unless the label of the cluster center is *i* (*i* = 1, 2, ∙∙∙, *k*), the labels of other pixels are initialized to −1.

2. Iterative clustering process.

Each pixel belongs to the cluster center closest to it. The search range of the cluster center is 2*S* × 2*S*, instead of global search. After all pixels are assigned to the nearest cluster center, the *i*-th cluster center vector value is updated by the mean value of the vector values of all pixels belonging to the *i*-th cluster center. The iteration termination condition is set to the number of iterations *n* (Equation (2)).

(2) $$n\leq n_{t},$$

where *n_t_* is the preset number of iterations.

In the iterative clustering process, the distance between the pixel and the cluster center is measured by weighted Euclidean distance. The distance calculation formula is as follows:

(3) $$D^{\prime }=\sqrt{{\left(\frac{d_{c}}{N_{c}}\right)}^{2}+{\left(\frac{d_{s}}{N_{s}}\right)}^{2}},$$

where $d_{c}=\sqrt{{\left(l-l_{i}\right)}^{2}+{\left(a-a_{i}\right)}^{2}+{\left(b-b_{i}\right)}^{2}}$ and $d_{s}=\sqrt{{\left(x-x_{i}\right)}^{2}+{\left(y-y_{i}\right)}^{2}}$; (*l, a, b, x, y*) is vector value of pixel(*x, y*); *N_c_* and *N_s_* are weighting factors.

**Figure 1:**
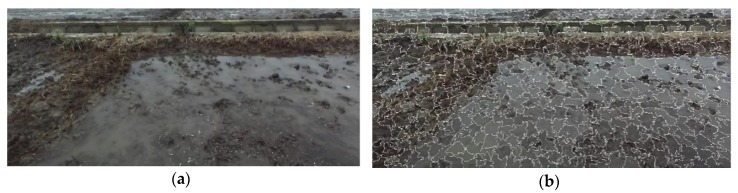
Effect of the simple linear iterative clustering algorithm (SLIC) segmentation process: (**a**) paddy field image; (**b**) SLIC superpixel segmentation for (**a**).

SLIC superpixel segmentation for a paddy field image (Figure 1a) can generate compact, nearly uniform superpixels (Figure 1b), and the algorithm runs fast.

### 2.2. Paddy Field Superpixel Features Extraction

For each superpixel, extract 9-dimensional color features and 10-dimensional texture features to form a 19-dimensional feature vector ***ν****_j_* (Equation (4)).

(4) $$v_{j}=\left(\mu_{jr},\mu_{jg},\mu_{jb},\mu_{jh},\mu_{js},\mu_{jv},\mu_{jm},\sigma_{jh},\sigma_{js},\sigma_{jv},h_{j1},h_{j2},h_{j3},h_{j4},h_{j5},h_{j6},h_{j7},h_{j8},h_{j9}\right),$$

where *j* is *j*-th superpixel; (*μ_jr_*, *μ_jg_*, *μ_jb_*, *μ_jh_*, *μ_js_*, *μ_jv_*) are the RGB three-channel mean and the HSV (Hue, Saturation, Value) three-channel mean of all pixels in the superpixel; (*σ_jh_*, *σ_js_*, *σ_jv_*) are HSV three-channel variance of all pixels in the superpixel; *μ_jm_* is the mean of the gradient amplitude of all pixels in the superpixel; (*h_j_*_1_, *h_j_*_2_, *h_j_*_3_, *h_j_*_4_, *h_j_*_5_, *h_j_*_6_, *h_j_*_7_, *h_j_*_8_, *h_j_*_9_) is 9-dimensional vector based on pixel gradient direction histogram in superpixel.

#### 2.2.1. Color Features Extraction

Color features are statistical characteristics of pixel values in superpixels based on RGB and HSV color spaces, which are obtained by calculating the RGB three-channel mean, HSV three-channel mean, and HSV three-channel variance of all pixels in the superpixel. The HSV color space is used because the HSV color space [22] has low sensitivity to natural light changes. The HSV three-channel variance calculation formula is as follows:

(5) $$\sigma^{2}=\sum {\left(z-\mu \right)}^{2},$$

where *z* is the HSV three-channel value of pixel (*x*, *y*) in superpixel; *μ* is mean of HSV three channels of all pixels in superpixel; *p*(*z*) is probability that pixel value is *z* in superpixel.

**Figure 2:**
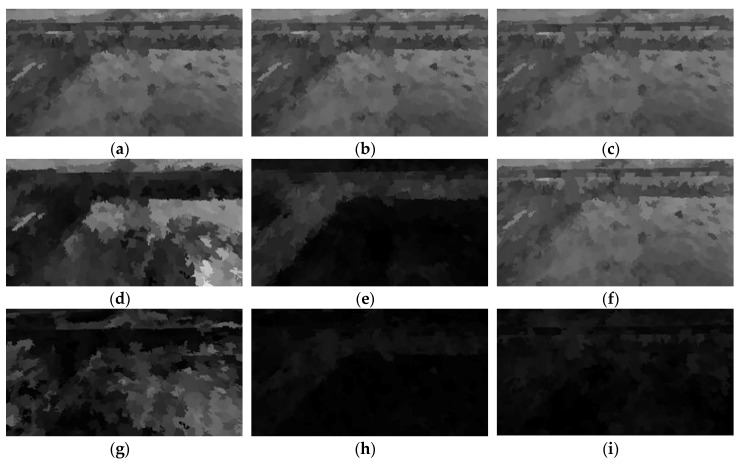
Graphic representation of superpixel color features: (**a**) R mean feature; (**b**) G mean feature; (**c**) B mean feature; (**d**) H mean feature; (**e**) S mean feature; (**f**) V mean feature; (**g**) H variance feature; (**h**) S variance feature; (**i**) V variance feature.

The super pixel color feature maps are shown in Figure 2.

#### 2.2.2. Texture Features Extraction

Different from the color features, the texture features of the image characterize the pixel value distribution relationship between the pixel and its spatial neighborhood, and describe the surface properties of the object corresponding to the image area. The texture features extracted in this paper include two parts: One is the average gradient magnitude of all pixels in the superpixel, and the other is a 9-dimensional vector based on the pixel gradient direction histogram.

The calculation formula of the pixel gradient amplitude *G*(*x, y*) is as follows:

(6) $$G\left(x,y\right)=\sqrt{G_{x}{\left(x,y\right)}^{2}+G_{y}{\left(x,y\right)}^{2}},$$

where $G_{x}\left(x,y\right)=I\left(x+1,y\right)-I\left(x,y\right)$ and $G_{y}\left(x,y\right)=I\left(x,y+1\right)-I\left(x,y\right)$; *I*(*x, y*) is the gray value of the pixel(*x*, *y*).

In actual processing, when calculating the horizontal gradient *G_x_* and vertical gradient *G_y_*, Sobel edge operator [23] is used for convolution with image *I* (Equation (7)).

(7) $$G_{x}=\left[\begin{array}{ccc} -1 & 0 & +1\\ -2 & 0 & +2\\ -1 & 0 & +1 \end{array}\right]*I\;\mathrm{and}\;G_{y}=\left[\begin{array}{ccc} +1 & +2 & +1\\ 0 & 0 & 0\\ -1 & -2 & -1 \end{array}\right]*I.$$

**Figure 3:**
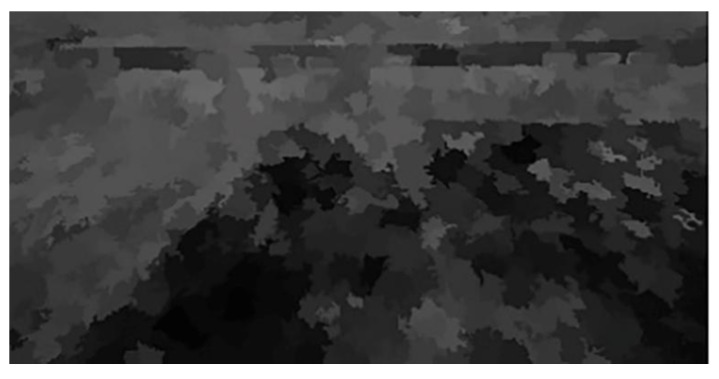
Graphic representation of superpixel gradient amplitude mean feature.

The superpixel gradient amplitude mean is shown in Figure 3.

The second part of the texture feature is a 9-dimensional vector based on pixel gradient direction histogram in superpixel. The gradient direction value of the pixel (*x*, *y*) is shown below:

(8) $$\alpha \left(x,y\right)=\tan^{-1}\left(\frac{G_{x}\left(x,y\right)}{G_{y}\left(x,y\right)}\right),$$

where *α*(*x*, *y*) is the value of the pixel(*x*, *y*) gradient direction, and the range is 0–360°.

**Figure 4:**
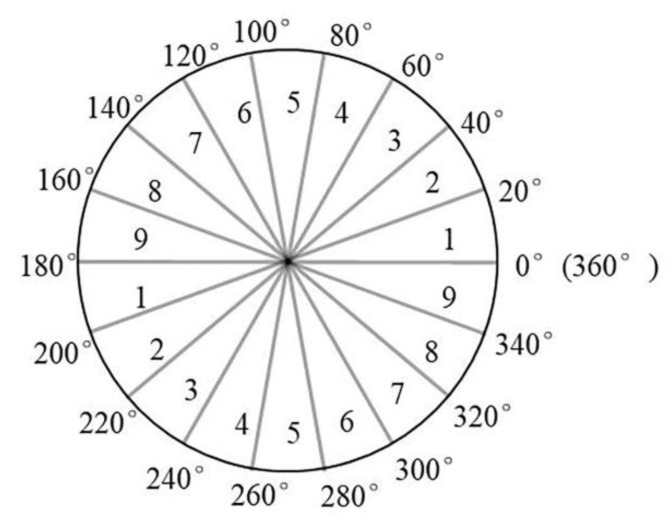
Block diagram of 0–360° angle.

Construct a 9-dimensional texture feature vector with the following rules:

1. 0–360° degree is evenly divided into 9 parts (Figure 4), and each part corresponds to a value of a 9-dimensional feature vector. Initialize each value of the 9-dimensional vector to 0.
2. Traverse all the pixels in the superpixel, and calculate the weighted value in the corresponding angle area according to the gradient direction value of the pixel. That is, when the gradient direction value*α*(*x*, *y*) of the pixel(*x, y*) satisfies:

(9) $$\alpha \left(x,y\right)\in p_{l},$$

where *p_l_* is the *l*-th angle range, *l* = 1,2,3, ∙∙∙, 9.

Calculate the weighting value of the vector value *h_l_* corresponding to *p_l_*, and use the pixel amplitude value as the weighting coefficient:

(10) $$h_{l}+=G\left(x,y\right)\times 1$$

### 2.3. Paddy Field Ridge Recognition Based on Support Vector Machine

#### 2.3.1. Obtaining and Processing of Training Samples for SVM

The training sample set in this paper consists of manually labeled superpixels. The 19-dimensional feature vector of the superpixels is used as input for the training data of the SVM model. The training label is −1 and 1. After the superpixel segmentation of collected paddy field images, the software is designed by OpenCV library, and the superpixels are marked by clicking the mouse. The superpixels of the ridge area are defined as positive sample (1), while the superpixels of the non-ridge area are defined as negative sample (−1). The superpixels are derived from twenty paddy field pictures collected in different paddy fields at different times of the day, and annotated to obtain 1266 positive samples and 2749 negative samples, thereby the training sample set (***ν****_j_*, *m_j_*) is obtained and *j* = 1,2,3,∙∙∙,4015, *m_j_* ∈ (−1, 1).

#### 2.3.2. Ridge Recognition SVM Model Training

The goal of the paddy field ridge recognition SVM model training is to find a hyperplane that can divide the two types of samples of ridge field and non-ridge field and maximize the interval between the two types of samples. The hyperplane can be expressed by the following linear equation:

(11) $$\omega^{T}v+b=0,$$

where ***ω*** is the normal vector of the hyperplane and determines the direction of the hyperplane; ***b*** is the displacement term and determines the distance between the hyperplane and the origin; ***v*** is the training data set.

In order to maximize the interval between the two types of samples, the SVM needs to solve the following secondary optimization problem:

(12) $$\begin{array}{l} \min\frac{1}{2}{\left\|\omega \right\|}^{2}\\ s.t.\;y_{j}\left(\omega^{T}v_{j}+b\right)\geq 1 \end{array}$$

After comparing the radial basis kernel function, polynomial kernel function and linear kernel function, the linear kernel function is selected as the kernel function of the SVM model because the classification results of the linear kernel function are better.

In addition, a cross-validation method is used to avoid SVM overfitting the training samples, and that is, the training samples are randomly divided into two parts of 9:1; 90% of the samples are used as model training samples for training the SVM model, and 10% of the samples are used as the validation set. It is used to verify the accuracy of the model, and the model with the highest accuracy in the validation set is used as the output model.

In the process of model training, marked positive and negative samples are used as model input, SVM model training parameters are set, linear kernel function of the model is selected, and iterative training was conducted. Finally, the optimal SVM model is selected by verifying the data set.

The linear kernel function is as follows:

(13) $$\kappa (xi,xj)=xi^{T}xj.$$

#### 2.3.3. Application and Verification of Ridge Recognition SVM Model

**Figure 5:**
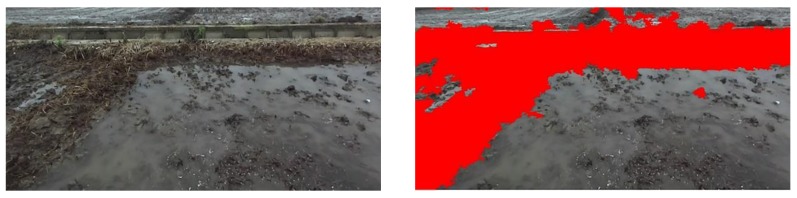
Classification results of paddy field with horizontal ridge at 9:00 a.m. in Songjiang District, Shanghai.

After SLIC superpixel segmentation, features are extracted for each superpixel, and a trained SVM model is loaded to predict each superpixel type. Figure 5 shows the effect of field classification recognition, where the red area indicates the field ridge. It can be seen from the classification results that the superpixels of the paddy field are basically accurately classified.

## 3. Experiment Results and Analysis

The algorithm processor is NVIDIA Jetson TX2 Processor @2.0 GHz CPU, 8.0 GB memory, and 32 GB data storage (NVIDIA, Santa Clara, CA, USA). The software used is the Ubuntu16.04 operating system (An open sourse free oeperatingn system). In the superpixel segmentation and SVM classification stages, the Superpixel SLIC and SVM functions from OpenCV, a computer vision open source library, are used.

The images were collected at 9:00 a.m. and 3:00 p.m. respectively in Songjiang District, Shanghai, and at 4:00 p.m. in Pudong District, Shanghai. Among the images taken in Songjiang District and Pudong District, 50 paddy field images containing ridges were selected. Therefore, there are about a dozen images at each time point, and each image can generate 500 superpixels, so the number of training samples at each time point is several thousand.

**Figure 6:**
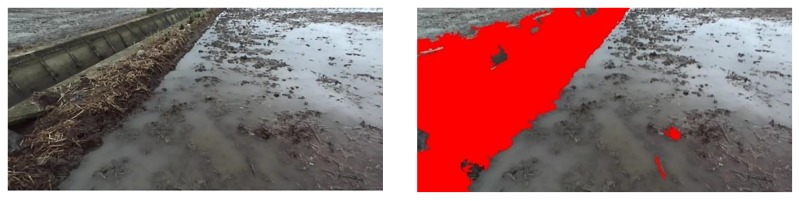
Classification results of a paddy field with a vertical ridge at 9:00 a.m. in Songjiang District.

**Figure 7:**
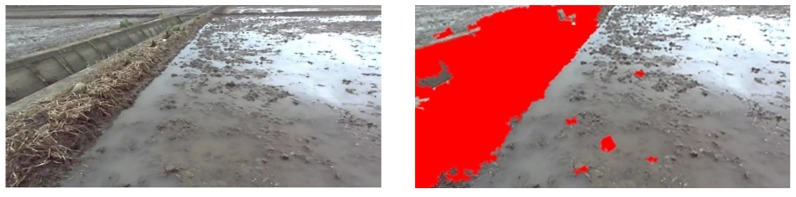
Classification results of a paddy field with a vertical ridge at 3:00 p.m. in Songjiang District.

When the Yanmar VP6E paddy field broadcast machine is traveling at a speed of 0.8 m/s, the ZED camera (Stereolabs, San Francisco, California, USA) collects paddy field pictures in real time, and Jetson TX2 completes the task of identifying a paddy field ridge in 0.6 s. For the paddy field in Songjiang District at different times, the field ridge recognition after SVM classification is shown in Figure 6 and Figure 7. Combined with the classification result graph of Figure 5, it can be seen that the algorithm used can well identify different types of field ridges in paddy fields at different times in Songjiang District.

**Figure 8:**
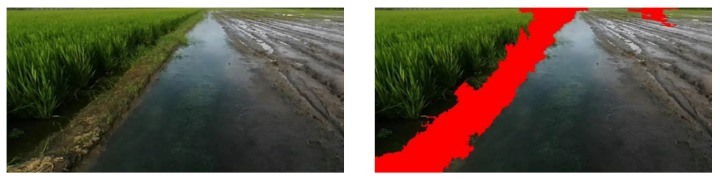
Classification results of a paddy field with a vertical ridge at 4:00 p.m. in Pudong District.

In order to verify that the field recognition algorithm has a certain generalization ability, this algorithm is applied in the paddy field at 4:00 p.m. in Pudong District, Shanghai (Figure 8). The classification result shows that the algorithm can effectively detect paddy field ridges in different paddy fields.

The F1 score [24] is used as the evaluation index of the paddy field ridge recognition algorithm. Among the pictures taken in Songjiang District and Pudong District, 50 paddy field pictures containing ridges were selected. The classification results of superpixels were compared with the results of actual manual labeling, and the accuracy and recall of superpixel recognition were also counted. 50 paddy field images were divided into 21,875 superpixels after superpixel segmentation. After manual labeling, the number of positive samples (superpixels in the ridge field) was 3365, and the number of negative samples (superpixels in the non-ridge field) was 18,510.

As can be seen from Table 1, the proposed algorithm can accurately segment the ridge field and non-ridge field in the paddy field, and the F1 score index reaches 90.7%, thereby the effectiveness of the algorithm is verified.

The route is the information that directly guides the automatic driving of agricultural machinery. After classifying a paddy field, therefore, it is necessary to further extract the boundary of the ridge field to use it as the route for agricultural machinery.

**Figure 9:**
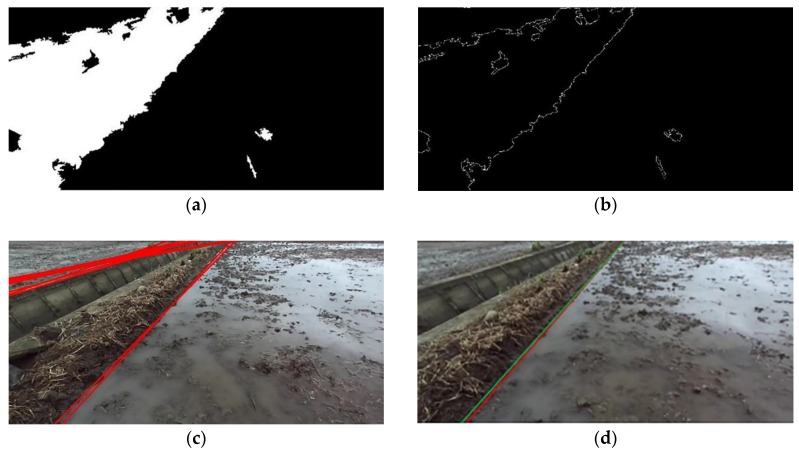
Graphic representation of farmland ridge boundary extraction: (**a**) binary map; (**b**) canny edge map; (**c**) Hough transform detection map; (**d**) boundary extraction map.

Taking the paddy field classification result map (Figure 6) as an example, the process of field boundary extraction is explained. Based on the classification results of SVM, the paddy field image is binarized to obtain a binary map (Figure 9a). A canny edge detection operator [25] is used to detect all edges of the binary map, and the edge map is obtained (Figure 9b). Finally, the Hough transform is used to detect all the straight lines in the edge map (Figure 9c). Because the Hough transform often detects more than one straight line, select the straight line closest to the bottom center of the picture, that is, coordinates (640,720) as the boundary of the field (Figure 9d). It can be seen from the Figure 9 that the boundary of a ridge field can be effectively extracted.

As shown in Figure 9d, the green line is the manually labeled field boundary and the red line is extracted by the algorithm. The angle between the two lines is used as the criterion for judging the detection accuracy of the boundary line. Calculate the angle between two lines in the image coordinate system. Take the top left corner of the image as the origin, the horizontal axis as the *X*-axis, the vertical axis as the *Y*-axis. In this coordinate system, the angle between the red or green line and the *X*-axis can be calculated, and then the angle can be obtained. Count the angle between the red and green lines in the 50 paddy field pictures selected above, with an average value of 1.63° and a variance of 0.14(°)^2^. Therefore, the accuracy of the field boundary extracted by the proposed algorithm can meet the requirements of automatic navigation.

Jetson TX2 completes the task of identifying the paddy field ridge in one image in 0.6 s. The navigation line extraction time is within 0.2 s, so the total processing time of the algorithm to process a frame of image is within 0.8 s. Meanwhile, since the forward looking distance of the ZED camera can reach 10 m, and the travel speed of the paddy field broadcast machine is 0.8 m/s, the algorithm processing time of 0.8 s can meet the real-time detection requirements.

## 4. Conclusions

Aiming at the paddy field ridge detection problem, support vector machine classification is used instead of the traditional image segmentation algorithm to segment the ridge field and non-ridge field in the image, and the ridge boundary is extracted using Hough transform. On a NVIDIA Jetson TX2 hardware platform, the total running time of the proposed algorithm is within 0.8 s, thus effectively meeting the real-time requirements.

1. Preprocessing the SLIC superpixel segmentation of the original paddy field image effectively reduces the calculation amount of subsequent algorithm processing, and provides a large number of samples for the SVM model training. Only 20 pictures are needed to obtain thousands of samples, which solves the problem that machine learning algorithms require a large number of samples.
2. Considering that there are certain differences in color and texture between the ridge field and non-ridge field areas, a 9-dimensional color feature vector and a 10-dimensional texture feature vector are extracted during the feature extraction stage, making full use of image information to make up for the disadvantages of traditional image segmentation algorithms relying on picture color information.
3. Processing multiple farmland images in different time periods and different plots, the results show that the proposed algorithm can accurately segment the ridge field and non-ridge part of a paddy field, and the F1 score index reaches 90.7%.

## Figures and Tables

**Table 1:** Statistical analysis of the classification results.

Camera	TP	TN	FP	FN	Accuracy (%)	Recall (%)	F1 Score (%)
ZED	3100	18,137	373	265	89.3	92.1	90.7

where TP indicates a superpixel that is predicted to be 1, and is actually 1; TN indicates a superpixel that is predicted to be 0, and is actually also 0; FP is a superpixel that is predicted to be 1, and is actually 0; and FN is a superpixel that is predicted to be 0, and is actually 1.
